# *ARABIDOPSIS THALIANA HOMEOBOX GENE 1* controls plant architecture by locally restricting environmental responses

**DOI:** 10.1073/pnas.2018615118

**Published:** 2021-04-22

**Authors:** Mahwish Ejaz, Stefano Bencivenga, Rafael Tavares, Max Bush, Robert Sablowski

**Affiliations:** ^a^Cell and Developmental Biology Department, John Innes Centre, NR4 7UH Norwich, United Kingdom

**Keywords:** plant development, plant architecture, stem growth, *Arabidopsis*

## Abstract

The diversity and environmental plasticity of plant growth results from variations of repetitive modules, such as the basic shoot units made of a leaf, axillary bud, and internode. Internode elongation is regulated both developmentally and in response to environmental conditions, such as light quality, but the integration of internal and environmental signals is poorly understood. Here, we show that the compressed rosette growth habit of *Arabidopsis* is maintained by the convergent activities of the organ boundary gene *ARABIDOPSIS THALIANA HOMEOBOX GENE 1* (*ATH1*) and of the gibberellin-signaling *DELLA* genes. Combined loss of *ATH1* and *DELLA* function activated stem development during the vegetative phase and changed the growth habit from rosette to caulescent. Chromatin immunoprecipitation high-throughput sequencing and genetic analysis indicated that *ATH1* and the *DELLA* gene *REPRESSOR OF GA1-3* (*RGA*) converge on the regulation of light responses, including the *PHYTOCHROME INTERACTING FACTORS* (*PIF*) pathway, and showed that the *ATH1* input is mediated in part by direct activation of *BLADE ON PETIOLE* (*BOP1* and *BOP2*) genes, whose products destabilize PIF proteins. We conclude that an organ-patterning gene converges with hormone signaling to spatially restrict environmental responses and establish a widespread type of plant architecture.

## Significance

A major issue in plant biology is how plants are shaped by the interaction between internal genetic programs—for example, those that form boundaries between leaves and the stem—and environmental signals such as light quality, which induces stem elongation in shade conditions. In many plant species, stem growth is suppressed during the vegetative phase, resulting in a compact whorl of leaves called a rosette. We show that the rosette habit of *Arabidopsis* is conferred by a gene involved in organ boundary formation, together with gibberellin hormone signaling, both of which antagonize genes that mediate organ growth in response to light. In this way, a common type of plant architecture results from localized inhibition of environmentally responsive growth.

The modular development of plants is particularly visible in the shoot, which is composed of repeated units (phytomers) comprising a leaf, an internode, and an axillary meristem nestled between them (1, 2). Much of the diversity in plant form and the plasticity of plant growth in different environments can be explained by variations in the development of these repetitive modules, such as the extent of internode elongation or whether axillary meristems remain dormant or initiate new branches. Revealing the genetic basis for these variations is also key to understanding the domestication of crop plants and to further improve crop productivity.

A striking example of adaptive variation in shoot architecture is the rosette habit, in which very short internodes result in a compact whorl of leaves close to the ground. This architecture is believed to be an adaptation to grazing, drought, and cold environments (3, 4). During the reproductive phase, rosette plants often produce a long inflorescence stem, which is likely an adaptation to facilitate seed dispersal. A prominent instance of plant with a rosette habit is the model species, *Arabidopsis thaliana*. Despite extensive genetic and developmental studies, the genetic basis for this central aspect of the growth habit remains virtually unknown. An important clue is that vegetative internode elongation can be induced by interfering with phytochrome signaling, as seen in *phyA phyB* double mutants (5), *phyB phyD phyE* triple mutants (6), or *phyB bop2* double mutants (7). However, phytochromes control numerous processes throughout the shoot and, consequently, all of these mutant combinations have pleiotropic effects on plant growth.

A well-studied case of plasticity in shoot architecture is the shade avoidance response (SAR), which is triggered by an increase in far-red (FR) light reflected by neighboring plants. The increase in FR light initiates a coordinated developmental response that inhibits branching and prioritizes elongation of the main shoot axis during competition for sunlight (8). Although this response is coordinated across multiple shoot organs, it is spatially restricted. For example, during the vegetative phase of *Arabidopsis*, the SAR affects elongation of the hypocotyl and petiole and cell proliferation in developing leaves, but does not overcome the inhibition of internode elongation that maintains the rosette habit, in contrast to the strong internode elongation that was originally used to define the SAR in caulescent plants (8, 9).

In *Arabidopsis*, phytochrome B (phyB) is the main photoreceptor that initiates the SAR. In normal light conditions, phyB phosphorylates a class of helix–loop–helix transcription factors, the PHYTOCHROME INTERACTING FACTORS (PIFs), leading to their inactivation or degradation (10). Exposure to FR light inactivates phyB and stabilizes PIFs, which in turn promote gene expression required for organ elongation (11). PIFs are also regulated by hormone signals; for example, they are inhibited by DELLA proteins, which play a central role in gibberellin (GA) signaling. In this way, PIFs function as a hub to integrate environmental and hormonal signals in the regulation of plant growth (12, 13).

We hypothesized that to control internode elongation and maintain the rosette habit, a widely used and environmentally dependent mechanism, such as phytochrome signaling, would need to be modulated locally. A good candidate to provide local control of internode growth is the BELL-type homeodomain gene *ARABIDOPSIS THALIANA HOMEOBOX GENE 1* (*ATH1*). During the vegetative phase, *ATH1* is expressed throughout the shoot meristem, subapical region, and at the base of leaf primordia, but is repressed in the shoot apex during the transition to flowering, when growth of the inflorescence stem is activated (14, 15). Subsequently, *ATH1* is expressed at the basal boundaries of cauline leaves and floral organs, where it is required for correct differentiation of boundary tissues (14). When expressed from a constitutive promoter, ATH1 strongly inhibits growth of the inflorescence stem, suggesting that the expression of ATH1 across the vegetative shoot apex could have a role in repressing stem growth before flowering (14, 16). However, the loss-of-function *ath1-3* mutant has only a subtle effect on vegetative internode growth, which is enhanced in low-light conditions, suggesting that *ATH1* could interact with a light-activated pathway to repress vegetative stem development (14). Here, we investigated how *ATH1* interacts locally with environmental signals to promote the rosette growth habit.

## Results

**Fig. 1.**
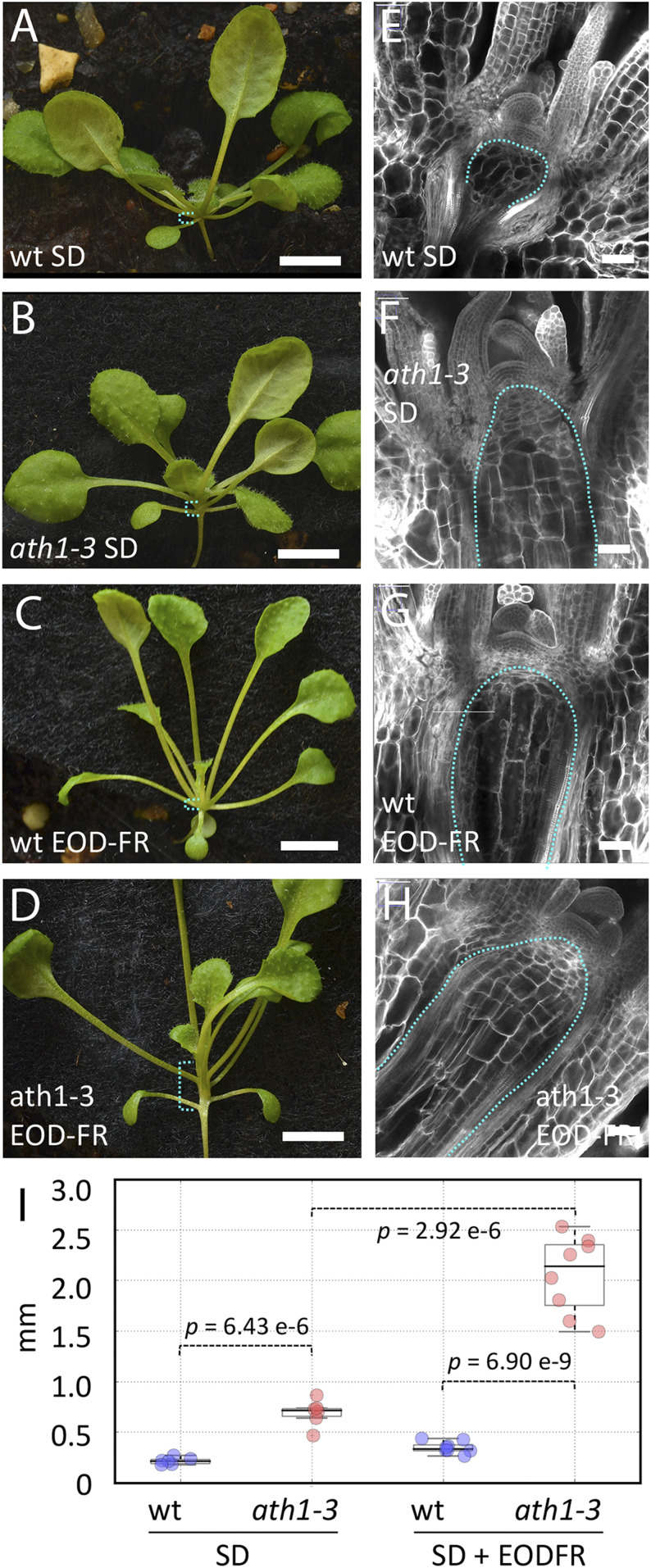
Loss of *ATH1* function makes rib meristem activity and internode growth responsive to far red light. (*A–D*) Wild-type Col (*A* and *C*) and *ath1-3* (*B* and *D*) seedlings grown for 21 short days (SD) without (*A* and *B*) or with EOD-FR light treatment (*C* and *D*); the brackets in cyan indicate the region where internode growth is repressed in the wild type. (Scale bars, 5 mm.) (*E–H*) Longitudinal optical sections though the shoot apex of seedlings corresponding to *A–D*, respectively; the dotted line in cyan surrounds the region corresponding to the rib meristem. (Scale bars, 50 µm.) (*I*) Boxplots showing the combined length of the first two vegetative internodes (millimeters) in seedlings as in *A–D*; individual data points for the wild type (Col) and *ath1-3* are shown in blue and red, respectively; *P* values are for Welch’s *t* test. Complete statistics (Shapiro–Wilk tests for normality, ANOVA, all pairwise *t* tests, power analysis) can be found in Dataset S4.

To test whether *ATH1* spatially restricts growth responses controlled by light signaling, we compared the loss-of-function *ath1-3* mutant with the corresponding wild type (Col-0), grown with or without end-of-day FR light treatment (EOD-FR), which induces the SAR (5). In short days without EOD-FR, *ath1-3* seedlings showed a small but significant elongation of vegetative internodes compared with Col-0 (Fig. 1 *A*, *C*, and *I*). To confirm that this phenotype resulted from activation of stem development, we imaged the rib meristem, where new stem tissues originate (17). Aligned cell files (Fig. 1 *E* and *G*) and incorporation of the nucleotide analog EdU (5-ethynyl-2′-deoxyuridine) (*SI Appendix*, Fig. S4*C*) showed that the rib meristem was active in *ath1-3* seedlings, but not in the wild type.

As expected (18), EOD-FR treatment induced petiole elongation in both Col-0 and in *ath1-3* (Fig. 1 *A*–*D*). In the wild type, EOD-FR light also induced elongation of rib meristem cells, although this was not sufficient to cause detectable internode elongation, presumably because these cells did not proliferate (Fig. 1 *E* and *F*). In contrast, EOD-FR–treated *ath1-3* showed enhanced rib meristem activity and clear internode elongation (Fig. 1 *G*–*I*). Vegetative internode elongation under EOD-FR reverted to wild type in *ath1-3* plants transformed with a genomic *ATH1* construct tagged with GFP (*pATH1:ATH1-GFP*) (*SI Appendix*, Fig. S1), confirming that the enhanced internode elongation was caused by loss of *ATH1* function. We conclude that *ATH1* inhibits rib meristem activity and vegetative internode elongation, and that in the absence of *ATH1*, stem growth is promoted as part of the SAR.

**Fig. 2.**
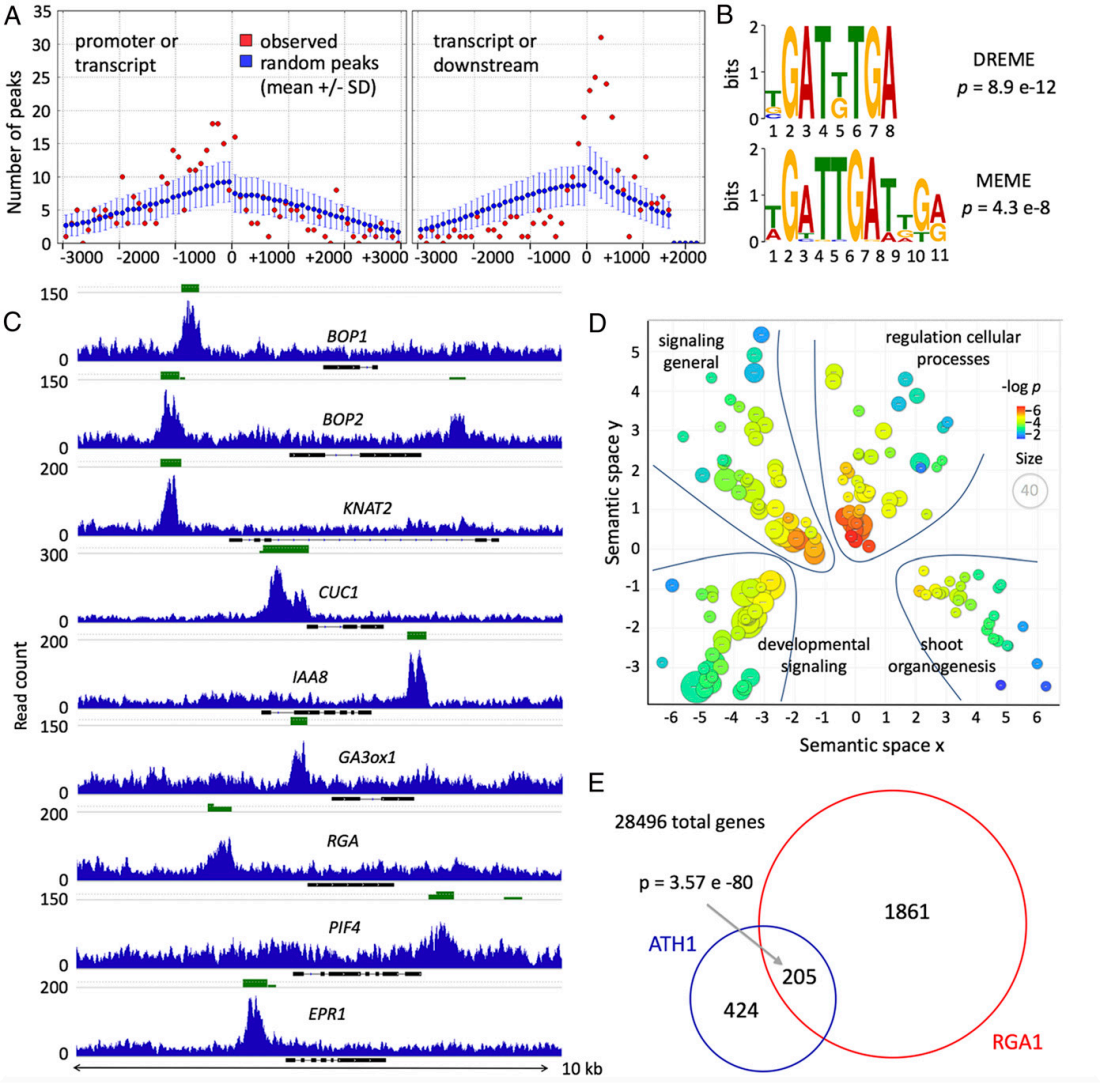
ATH1 binds in vivo to genes involved in meristem function, hormone and light signaling, many of which are also targeted by the DELLA protein RGA. (*A*) Enrichment of ATH1 binding sites within promoter and downstream regions compared with transcribed regions, for the 629 high-confidence ATH1 candidate targets (*SI Appendix*, Table S1); red dots show the frequency of peak regions (overlap of peaks from three ChIP-seq replicates) centered at the indicated nucleotide positions relative to transcript start or end; blue bars show how simulated peaks at random genome positions were distributed within the same set of high-confidence ATH1 targets; the observed ATH1 binding sites were significantly enriched in promoter and downstream regions (*P* < 0.05) and depleted in transcribed regions (*P* < 10^−3^; *P* values calculated by bootstrapping with 10,000 simulations). (*B*) Enrichment for sequence motifs detected using the MEME suite (20). Motifs within 75 nt of the center of ATH1 peaks analyzed in *A*, at least 6 bp long and with *P* > 0.001. (*C*) Representative raw ChIP-seq peaks (of three ChIP-seq replicates with similar peaks) within a 10-kb genomic window, for examples of genes involved in shoot organogenesis (*BOP2*, *KNAT6*, *KNAT2*, *CUC1*), hormone signaling (*IAA8*, *Ga3ox1*, *RGA*), or light signaling (*PIF4*, *EPR1*); black bars and lines correspond to exons and introns, respectively; green bars above the peaks show the overlapping peak regions detected in the three biological replicates. (*D*) Semantic clustering of GO categories enriched in the set of 629 ATH1 targets (Dataset S1); the diameter and color of each circle reflect the size and *P* value, respectively, for individual GO terms; four broad groups of GO terms are marked (see Dataset S3 for list of terms and genes in each group). (*E*) ATH1 ChIP-seq targets (blue) and published RGA targets (41) show a significantly larger overlap than expected by chance (two-tailed Fisher’s exact test).

*ATH1* encodes a BELL-type DNA-binding homeodomain protein, so it is expected to function by directly regulating gene expression. To gain insight into how *ATH1* might interact with light-responsive pathways, we used chromatin immunoprecipitation followed by high-throughput sequencing (ChIP-seq) to reveal genes targeted by ATH1. To tag genomic sites bound by ATH1, we used *ath1-3* transformed with the functional *pATH1:ATH1-GFP*, which was expressed in the vegetative apex similarly to endogenous *ATH1* (14) (*SI Appendix*, Fig. S1). ChIP-seq peaks were selected using MACS2 (19) and filtered for a false-discovery rate lower than 0.001 in all three *pATH1:ATH1-GFP* replicates, but in none of the wild-type replicates. Candidate target genes were associated with peaks within 3-kb upstream to 1.5-kb downstream of their coding sequences, with no other intervening genes. With these criteria, we selected 629 genes as candidate ATH1 targets (Dataset S1). As expected for a transcription factor, ATH1 frequently bound near the start or end of transcribed sequences, as seen in a histogram of peak locations (Fig. 2*A*) and in individual target genes relevant to this work (Fig. 2*C*). MEME-ChIP analysis (20) revealed that the regions bound by ATH1 were enriched for a motif bound by other plant homeodomain proteins (WOX13, ATHB12) and enriched in ChIP-seq peaks for REPLUMLESS, which is a BELL-type transcription factor related to ATH1 (21) (Fig. 2*B*).

Gene ontology (GO) analysis (22) of target genes showed a strong enrichment for DNA binding, suggesting that *ATH1* is part of a large transcriptional regulatory network (Dataset S2). Clustering of GO terms by semantic similarity (23) resulted in four main clusters, of which two corresponded to broad signaling and regulatory functions (Fig. 2*D* and Dataset S3). The two more specialized clusters corresponded predominantly to developmental signaling and shoot organogenesis; these two clusters overlapped by 21% and together corresponded to 65% of the genes that showed GO enrichment. The developmental signaling cluster included genes involved in hormone synthesis, transport, and signal transduction, predominantly for auxin (*ETTIN*, *IAA8*, *IAA9*, *IAA18*, *GH3.9*, *PIN7*, *LAX1*) (24–28) and GA (*GA4*, *RGA*, *GA2ox6*) (29–31). The shoot organogenesis cluster included genes that function closely with *ATH1* based on genetic evidence (*BOP1*, *BOP2*, *KNAT6*) (32, 33), genes involved in shoot meristem development (*KNAT2*, *KNAT6*, *BAM3*) (34, 35) and additional genes involved in organ boundary development (*CUC1*, *LSH4*, *LOB*) (36–38). Overall, the ChIP-seq experiments indicated that *ATH1* functions to a large extent by orchestrating hormonal signaling and the activity of other regulatory genes involved in early shoot organogenesis.

The increased rib meristem activity in *ath1-3* was reminiscent of the activation of stem development by treatment of rosette plants with GA (39). Moreover, the SAR induces petiole elongation partly through GA biosynthesis and the consequent degradation of DELLA proteins (40). To check whether ATH1 and DELLA proteins control similar processes to repress internode elongation, we overlapped the ATH1 candidate targets with target genes of the DELLA protein REPRESSOR of ga1-3 (RGA), identified in a comparable ChIP-seq analysis (41). The overlap of ChIP-seq targets for ATH1 and RGA was considerably higher than expected by chance (Fig. 2*E*), supporting the idea that these proteins control similar downstream processes. The overlapping set of targets also indicated reciprocal interactions, with ATH1 binding to *RGA* and, conversely, RGA binding to genes that are closely associated with *ATH1* function, such as *BOP1*, *BOP2*, and *KNAT6* (32, 33).

**Fig. 3.**
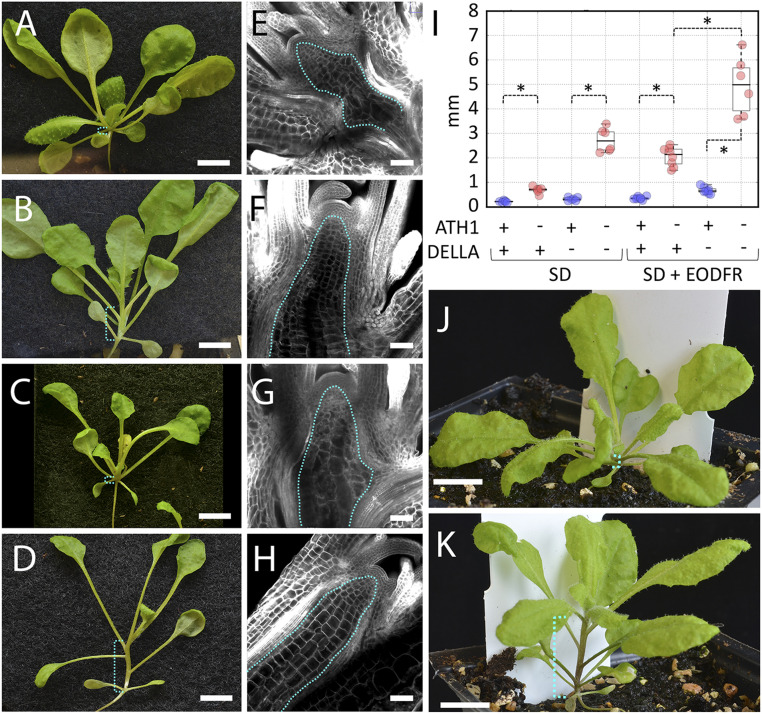
*ATH1* and *DELLA* genes converge on the control of vegetative internode elongation. (*A–D*) *global della* (*A* and *C*) and *ath1-3 global della* (*B* and *D*) seedlings grown for 21 short days without (*A* and *B*) or with EOD-FR light treatment (*C* and *D*); the brackets in cyan indicate where vegetative internodes develop. (Scale bars, 5 mm.) (*E–H*) Optical sections though the shoot apex of seedlings corresponding to *A–D*, respectively; the dotted cyan line surrounds the rib meristem. (Scale bars, 50 µm.) (*I*) Boxplots of internode length (combined length of the first two vegetative internodes) in seedlings with the genotypes and light treatments indicated (“+” and “–“ indicate wild type or mutant, respectively); individual data points for *global della* (*gd*) and *global della* combined with *ath1-3* are shown in blue and red, respectively; asterisks indicate significantly different means (*P* < 0.01, Welch’s *t* test); for a complete statistical analysis, see Dataset S4. (*J* and *K*) Representative *Arabidopsis* plants grown for 35 short days; the dotted brackets in cyan indicate the vegetative stem; (*J*) wild type (Col); (*K*) *ath1-3 global della* sextuple mutant. (Scale bars, 1 cm.)

To confirm whether the large overlap in ChIP-seq targets reflected a functional overlap, we next combined *ATH1* and *DELLA* mutants. To ensure complete loss of DELLA function, we used a background with mutations in all five *Arabidopsis DELLA* genes (*rga-t2*, *gai-t6*, *rgl1-1*, *rgl2-1*, and *rgl3-1*, called for simplicity the *global della* mutant) (13). The sextuple *ath1-3 global della* mutant showed a much clearer elongation of vegetative internodes than either *ath1-3* or *global della* (compare Fig. 1 *A*, *B*, and *I* with Fig. 3 *A*, *B*, and *I*). The enhanced phenotype cosegregated with *ath1-3* in the progeny of the sextuple mutant crossed to heterozygous *ath1-3* in *global della* background (12 of 12 plants with long internodes were homozygous for *ath1-3* and 6 of 6 short plants were heterozygous). Furthermore, we independently verified the phenotype caused by combined loss of *ATH1* and *DELLA* function using continuous treatment of *ath1-3* with GA to induce DELLA degradation, either by spraying (*SI Appendix*, Fig. S2) or by growth in medium with GA (see, for example, Fig. 5 *C* and *D* and *SI Appendix*, Fig. S3).

The internode elongation in the sextuple mutant was associated with full activation of the rib meristem, shown both by well-defined cell files below the shoot meristem (Fig. 3 *E* and *F*) and by incorporation of the nucleotide analog EdU (42) (*SI Appendix*, Fig. S4*D*), and was further enhanced by EOD-FR treatment (Fig. 3 *C*, *D*, and *I*), resulting in an extensive rib meristem (Fig. 3 *G* and *H*), comparable to what is seen in the *Arabidopsis* inflorescence stem (41). When the sextuple mutant was grown under short days without EOD-FR, the elongation of vegetative internodes became more obvious as the plants matured, resulting in a full conversion from rosette to a caulescent habit before the inflorescence stem emerged (Fig. 3). We conclude that *ATH1* and *DELLA* genes function redundantly to repress growth of the vegetative stem and maintain the rosette habit.

**Fig. 4.**
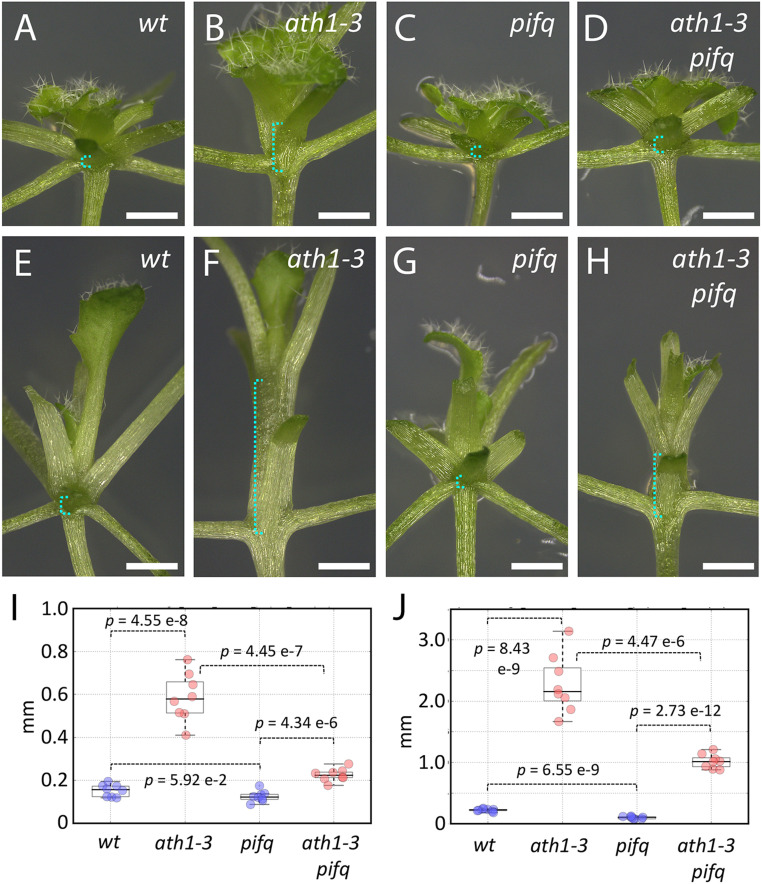
*PIF* genes promote elongation of vegetative internodes in *ath1-3*. (*A–H*) Col (*A* and *E*), *pifq* (*B* and *F*), *ath1-3* (*C* and *G*), and *ath1-3 pifq* (*D* and *H*) seedlings were grown for 21 short days without (*A*–*D*) or with (*E*–*H*) EOD-FR treatment, with older leaves dissected to expose the vegetative nodes; brackets in cyan indicate the distance between the first and third nodes measured in *I* and *J*. (Scale bars, 5 mm.) (*I* and *J*) Boxplots of internode length (combined length of the first two vegetative internodes) in seedlings with the genotypes indicated, grown for 21 d without (*I*) or with (*J*) EOD-FR treatment; individual data points in blue and red correspond, respectively, to control genotypes (wt Col, *pifq*) and the corresponding genotypes in *ath1-3* background; *P* values are for Welch's *t* test. Dataset S4 contains further statistical analysis.

The set of 205 loci bound by ATH1 and RGA in the ChIP-seq experiments included multiple genes in the PIF pathway, which controls shoot growth in response to environmental signals, such as light quality and ambient temperature (43). The shared ChIP-seq targets encoded proteins involved in multiple steps of the PIF pathway, including one of the PIFs (PIF4), control of PIF activity (phyB, RGA) (12) or stability (RGA, BOP1, BOP2) (7, 12, 44), and downstream transcriptional responses (ATHB2, PIL1) (45, 46). To confirm whether *PIF* genes participate in the vegetative stem elongation seen in *ath1-3*, we combined loss of *ATH1* and *PIF* function. Considering that phyB has been implicated in the control of internode elongation (5–7), we used the quadruple *pifq* mutant (*pif1-1*, *pif3-3*, *pif4-2*, *pif5-3*) (47), with loss-of-function of the four *PIF* genes implicated in phyB signaling (43). Under normal light conditions, *pifq* eliminated the mild internode elongation seen in *ath1-3*, while under EOD-FR, *pifq* reduced internode elongation by about 50% (Fig. 4). Thus, *PIF* activity is important for the internode elongation seen in *ath1-3*, although under shade conditions *ATH1* must also antagonize genes that promote internode growth independently of *PIF1*, -*3*, -*4*, and -*5*.

**Fig. 5.**
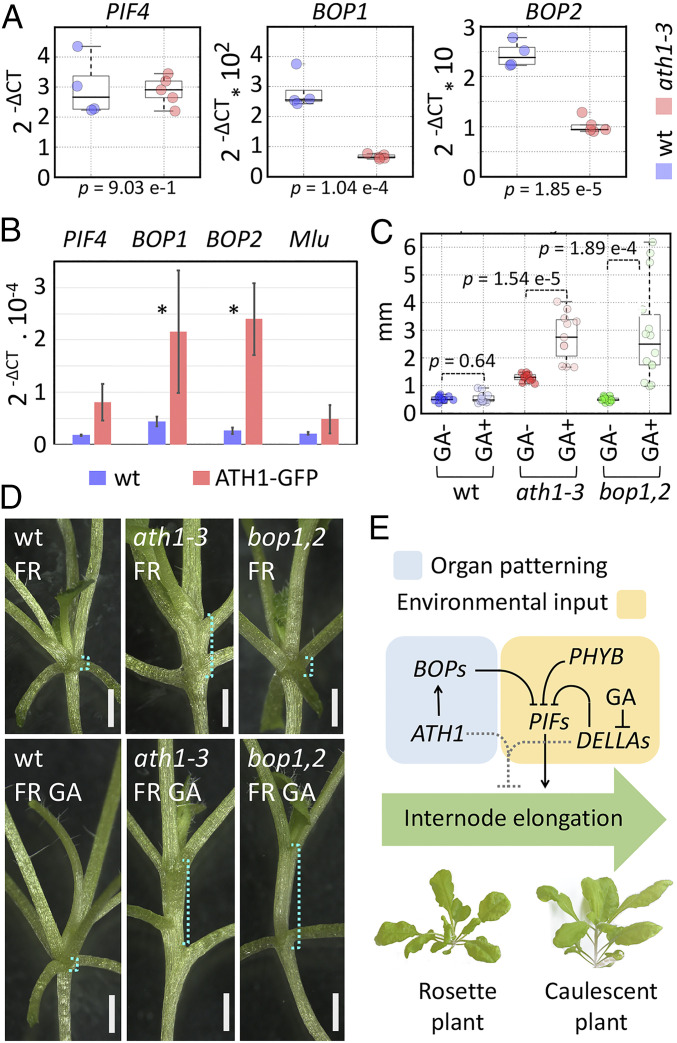
*BOP1* and *BOP2* are directly activated by ATH1 and inhibit internode elongation redundantly with GA signaling. (*A*) Expression of *PIF4*, *BOP1*, and *BOP2* in 10-d-old wild-type (blue) and *ath1-3* (red) seedlings, measured by qRT-PCR, with ΔCT relative to the PP2A internal control; *P* values are for Welch’s *t* test. Dataset S6 contains further statistical analysis. (*B*) Binding of ATH1-GFP to *PIF4*, *BOP1*, *BOP2*, and *Mlu* (negative control) shown by ChIP-qPCR; ΔCT values are relative to input DNA (equally diluted for all assays); bars and error bars show the average and SD for three biological replicates; asterisks indicate *P* values lower than 0.05 (Welch’s *t* test; raw data and statistical analysis in Dataset S7). (*C*) Length of the first internode in wild-type (blue), *ath1-3* (red), and *bop1-3 bop2-1* (green) seedlings grown for 14 d under short day EOD-FR conditions on medium with 10 µM GA4 (pale color) or without GA (dark color); *P* values are for Welch’s *t* test; see additional statistics in Dataset S4. (*D*) Representative wild-type, *ath1-3*, and *bop1-3 bop2-1* seedlings grown as in *C*; cyan brackets show the regions measured in *E*. (Scale bars, 1 mm.) (*E*) Summary diagram of the regulatory interactions between *ATH1*, *BOP*, *DELLA*, and *PIF* genes in vegetative internodes; arrows and blunted lines indicate positive and negative interactions, respectively; dotted lines represent additional, *PIF*-independent functions of ATH1 and DELLA proteins in repressing internode elongation.

Of the multiple links between ATH1 and *PIF* signaling suggested by the ChIP-seq data, we next explored two. First, we tested whether ATH1 controls *PIF4* transcription, using qRT-PCR. In line with the weak binding of ATH1 to *PIF4* in ChIP-seq experiments (Fig. 2), ChIP-PCR did not confirm significant binding (Fig. 5*B*) and we could not detect significant differences in *PIF4* expression in *ath1-3* (Fig. 5*A*). Thus, *PIF4* did not appear to be directly repressed by ATH1, although we cannot exclude a spatially or temporally restricted regulation. Second, we focused on *BOP1* and *BOP2*, whose protein products bind and destabilize PIF4 (7). The strong binding of ATH1 seen in the ChIP-seq experiments (Fig. 2*C*) was independently confirmed by ChIP-PCR (Fig. 5*B*) and qRT-PCR of seedlings grown for 14 short days showed reduced expression of both *BOP1* and *BOP2* in *ath1-3* compared with the wild type (Fig. 5*A*). Together, the ChIP-seq and expression data supported that ATH1 directly activates of *BOP1* and *BOP2* transcription.

Considering the finding that BOP proteins act as adaptors for a E3 ubiquitin ligase complex that targets PIF4 for degradation (7), the results above suggested that, like *DELLAs*, *ATH1* affects *PIF* function posttranscriptionally, albeit indirectly through *BOP* genes. To test to what extent *BOP* activity could account for the convergence of *ATH1* and *DELLAs* on the control of internode elongation, we next compared vegetative internodes in wild-type, *ath1-3*, and *bop1-3 bop2-1* mutants, with or without GA treatment, grown with EOD-FR treatment. Different from *ATH1*, loss of *BOP1,2* function was not sufficient to cause internode elongation, and neither was GA treatment alone (Fig. 5 *C* and *D* and *SI Appendix*, Fig. S2). However, GA strongly induced internode elongation in the *bop1,2* mutant (Fig. 5 *C* and *D*). Thus, as seen for *ATH1*, *BOP1* and *BOP2* function redundantly with *DELLA* genes to repress internode elongation induced by EOD-FR. Based on these results, combined with the evidence for direct activation of *BOP* genes by ATH1, we conclude that *BOP* genes function downstream of *ATH1* in the repression of internode elongation under shade avoidance conditions. However, *ATH1* and *DELLA* genes must also regulate processes independent of *BOP* genes to repress internode development under normal light conditions (Fig. 5*E*).

## Discussion

Here, we show that ATH1 and DELLA proteins converge to maintain the *Arabidopsis* rosette habit. Although the rosette habit is a prominent feature of *Arabidopsis* development, its regulation had eluded genetic analysis. The likely reason is the functional redundancy at multiple regulatory levels, both within gene families (*DELLA*, *PIF*, *BOP* genes) and across gene families (between *DELLA*s and *ATH1* or *BOP* genes). Nevertheless, the activation of vegetative internode elongation in the *ath1-3 global della* sextuple mutant, under normal growth conditions and with few other effects on plant architecture (Fig. 3), shows that a relatively simple genetic change can determine the difference between the rosette and caulescent growth habits.

Combined with published results (7), our data establish a molecular pathway through which ATH1 converge with DELLA proteins on the control of light-induced growth through the *PIF* pathway (Fig. 5*E*). A role for BOP proteins in mediating the role of *ATH1* in repressing light-induced internode growth is consistent with genetic evidence that *BOP* and *ATH1* genes form a functional module in inflorescence development (33, 48), and with the internode elongation seen in *bop2 phyB* and *bop1 phyB* double mutants grown in constant red light (7). However, in the absence of GA treatment, loss of *ATH1* function still caused a significant increase in internode growth not seen in *bop1-3 bop2-1*, therefore not all aspects of internode control by *ATH1* are mediated by *BOP* genes. Furthermore, *BOP* genes participate in a broader range of developmental processes than *ATH1*, including abaxial-adaxial leaf patterning and restriction of meristem identity (49–51). Thus, the available data indicate that although *BOP* and *ATH1* likely form a functional module, *ATH1* provides this module with specificity for the control of internode growth.

A similar convergence between a localized transcription factor and GA signaling has been recently described in rice (52), in which the Zn-finger transcription factor PREMATURE INTERNODE ELONGATION 1 (PINE1) represses internode elongation until the transition to flowering. In addition, *pine1* mutants show increased sensitivity of internodes to GA and premature internode elongation. As seen for *PINE1*, *ATH1* inhibits internode growth, is repressed during the floral transition, and blocks GA signaling in the internodes (i.e., loss of DELLA proteins cannot promote internode elongation unless ATH1 is also absent). Given that PINE1 and ATH1 are unrelated transcription factors, it appears that *Arabidopsis* and rice have evolved analogous mechanisms to control plant architecture through localized restriction of GA responses.

More generally, our results illustrate in plants how discrete developmental features can be produced by localized modulation of widely used signaling mechanisms, as seen for example for Wnt signaling in butterfly wings (53). Furthermore, the small genetic divergence seen here between rosette and caulescent growth is consistent with the rapid diversification of growth habits seen in different plant genera, for example *Lupinus* and *Brassica* (54). Specifically, in Brassicas human selection has established a rich variety of morphotypes, including parallel selection of compressed vegetative growth (55). In the future, it will be interesting to investigate whether differences in *ATH1* function and its interaction with GA signaling underlie changes in plant growth habit selected naturally or by humans.

## Materials and Methods

### Plant Genotypes.

Wild-type *A. thaliana* was the Columbia-0 (Col-0) accession. *ath1-3* (14) and the *global della* mutant (13) (*gai-t6*, *rga-t2*, *rgl1-1*, *rgl2-1*, *rgl3-1,* backcrossed twice into Col-0 background) were ordered from the Nottingham *Arabidopsis* Stock Centre. The *pifq* quadruple mutant (*pif1-1*, *pif3-3*, *pif4-2*, *pif5-3* in Col-0 background) (47) was kindly provided by Elena Monte, Center for Research in Agricultural Genomics, Barcelona, Spain. The *bop1-3 bop2-1* double mutant (Col background) (56) was sent by Veronique Pautot, Institut Jean-Pierre Bourgin, Paris, France; the GFP-RGA-Δ17 (57) and GFP-RGA (58) were provided by Tai-Ping Sun, Duke University, Durham, North Carolina.

To combine *ath1-3* with loss of *DELLA* or *PIF* function, *ath1-3* was crossed to *global della* or *pifq*, then backcrossed to *global della* or *pifq.* Heterozygous *ath1-3* plants in *global della* or *pifq* background were selected by PCR genotyping and selfed to produce fully homozygous plants. For genotyping, DNA was extracted as described previously (59), and PCR was performed using Q5 high fidelity Taq DNA polymerase (New England Biolabs) following the manufacturer's instructions with primers shown in Dataset S5. The genotyping was also confirmed by phenotypic analysis: *ath1-3* was tracked by defects in floral organ abscission (14), *global della* was scored based on floral organ defects, and *pifq* mutants were selected based on the short hypocotyls of dark-germinated seedlings.

### Plant Growth.

*Arabidopsis* seeds were imbibed and stratified at 4 °C for 4 d before planting on John Innes Centre *Arabidopsis* Soil Mix (Levington F2 compost with Intercept and 4-mm grit at a 6:1 ratio) in growth cabinets (Sanyo MLR-351), with ∼100 µE/m^2^ light from LED arrays (NVC Lighting; NL/18/LED/T8/4/840, 800 lm, 4,000 K), with cycles of short days (8-h light/16-h dark) or long days (16-h light/8-h dark). EOD-FR used short-day cycles, with a 15-min pulse of FR light (Rapid LED; CREE LED array, peak wavelength 730 nm, 250 µE/m^2^) at the end of an 8-h light period.

### Plasmid Construction and Transformation.

To produce *pATH1::ATH1-GFP*, 3.2 kb of the *ATH1* promoter was amplified in two fragments with primers OL278-OL279 and OL280-OL281 (Dataset S5). The *ATH1* coding sequence was ordered as a synthetic fragment (ThermoFisher Scientific, GeneArt) to remove BsaI sites. The promoter, gene, nopaline synthase (NOS) terminator, and GFP were assembled by Golden Gate cloning (60). The final *pATH1::ATH1-GFP* assembly was verified by sequencing and inserted into pPZP222 (61) for transformation of *ath1-3* using the floral-dip method (62). Sixteen independent transgenic lines were analyzed for complementation of *ath1-3* and GFP expression. For subsequent ChIP-seq experiments, a single line was selected, which fully complemented the *ath1-3* phenotype and carried one copy of the transgene, estimated by quantitative real-time PCR (iDNA Genetics).

### ChIP.

For ChIP, *pATH1:ATH1-GFP* or Col-0 controls were grown for 3 wk under short days (8-h light/16-h dark) and harvested at Zeitgeber time (ZT) 6; 2 to 3 g of seedlings were used per biological replicate, with leaves and roots were removed just before fixation. ChIP was performed as described previously (63), except that for ChIP-qPCR the IP buffer included salmon sperm (Sigma; DNA 0.5 mg/mL) and incubation with anti-GFP µMACS microbeads was for 1 h on ice. Generation of CHIP-seq libraries and analysis were as described previously (21). To screen for enriched motifs, MEME-ChIP was used in discriminative mode (https://meme-suite.org/tools/meme-chip) (20), comparing peak sequences with a 10-fold larger control set of random peaks, as described previously (21). Raw and processed ChIP-seq data have been deposited in the National Center for Biotechnology Information (NCBI) Gene Expression Omnibus GEO (64).

### Gene-Expression Analysis.

Plants were grown for 3 wk on one-half MS medium without sucrose (62) under the conditions described above. Fifteen to 20 seedlings were pooled per biological replicate. Total RNA was extracted using RNAeasy Qiagen plant mini extraction kit followed by DNase treatment using Turbo DNase free kit (Invitrogen). Less than or equal to 5 μg of total RNA was then reverse-transcribed using oligo(dT) (20-mer) and Super- Script IV reverse-transcriptase (Invitrogen) according to the manufacturer’s instructions. Relative expression analysis was performed on 1:10 diluted cDNA, SYBR GREEN^R^ JumpStart Taq ReadyMix (Sigma) and the primers listed in Dataset S5, using the LightCycler 480 Software (Roche; v1.5). PP2AA3 (At1g13320) was used as an internal constitutive control. Each treatment had at least four biological replicates, each of which corresponded to the average of four technical replicates. The raw data are included in Dataset S6.

### Immunoblots.

Wild-type (Landsberg-*erecta*), GFP-RGA-Δ17 (57), and GFP-RGA (58) plants were grown on GM medium with 10 μM GA or with EtOH control under short day EOD-FR for 14 d. Twenty-five seedlings per sample were used for protein extraction and immunoblotting as described previously (65), using 4 to 20% Mini-Protean TG gels (Bio-Rad) and PVDF membrane (Trans-Blot Turbo Mini, Bio-Rad); before blocking, the membranes were stained with Ponceau S to confirm equal loading. The blots were probed with rabbit anti‐GFP polyclonal antibody (AbCam ab6565, diluted 1:2,500) and Goat Anti-Rabbit IgG H&L (HRP) (Abcam ab205718, diluted 1:5,000) and bands were detected by ImageQuant LAS 500 using Clarity Western ECL Substrate (Bio-Rad). Similar results were obtained in two independent experiments, each with two biological replicates.

### Confocal Imaging.

Pseudo-Schiff propidium iodide (mPS-PI) staining were as described previously (66). GFP was imaged in cleared apices by the ClearSee method (67). For EdU labeling, 10-d-old seedlings were used. To facilitate diffusion into the rib zone, the cotyledons were cut close to the shoot apex and the seedlings were immediately incubated in direct contact with growth medium containing 10 μm EdU (Invitrogen, cat no: A10044) for 6 h. EdU detection was combined with mPS-PI staining, as described previously (66). Confocal imaging was performed with a Zeiss LSM780 microscope with (a 20×/0.75 long-working distance) objectives (0.42 × 0.42 × 0.50-μm resolution).

### Internode Measurements.

Internode elongation was measured as the distance between the base of cotyledon petioles and the base of the petioles of the second pair of true leaves in 21-d-old seedlings, or to the first pair of true leaves for seedlings grown for 14 d on plates. Distances were measured on seedling images relative to the corresponding scale images using Fiji (68).

### Statistical Analysis.

For statistical analysis and plotting graphs, functions were used from Numerical Python (https://numpy.org), Scientific Python (https://www.scipy.org), and MatPlotLib (https://matplotlib.org). For each treatment, normal distribution was tested using the Shapiro–Wilk test. Equality of means across multiple treatments and in pairwise comparisons was tested by one-way ANOVA and Welch’s *t* test, respectively. Effect sizes were measured using Cohen’s *d*-number. Power analysis was performed with the G*Power software (69), with a significance level of 0.01 and minimum power of 0.95. Raw values, descriptive statistics, *P* values, effect sizes and power analysis for the data used in each figure are listed in Dataset S4. In all figures with boxplots, boxes extend from the lower to the upper quartile, with a line marking the median, and whiskers extend to 1.5 times the interquartile range.

## Supplementary Material

Supplementary File

Supplementary File

Supplementary File

Supplementary File

Supplementary File

Supplementary File

Supplementary File

Supplementary File

## Data Availability

Raw and processed ChIP-seq data are available at the NCBI’s Gene Expression Omnibus database, https://www.ncbi.nlm.nih.gov/geo (accession no. GSE157332) (70). All other data are available within the main text and supplementary information.
