# Supplementary material for: ARABIDOPSIS THALIANA HOMEOBOX GENE 1 controls plant architecture by locally restricting environmental responses
Source: Proc Natl Acad Sci U S A. 2021 Apr 22;118(17):e2018615118. doi: 10.1073/pnas.2018615118 (PMC8092594; doi:10.1073/pnas.2018615118)
Supplement: Supplementary File [file pnas.2018615118.sapp.pdf]

PNAS
www.pnas.org

Supplementary Information for

ARABIDOPSIS THALIANA HOMEOBOX GENE 1 controls plant architecture by locally restricting environmental responses

Mahwish Ejaz, Stefano Bencivenga, Rafael Tavares, Max Bush & Robert Sablowski*

*Corresponding author
Email: robert.sablowski@jic.ac.uk

This PDF file includes:

Figures S1 to S4
Legends for Datasets S1 to S7
Supplementary references

Other supplementary materials for this manuscript include the following:

Datasets S1 to S7

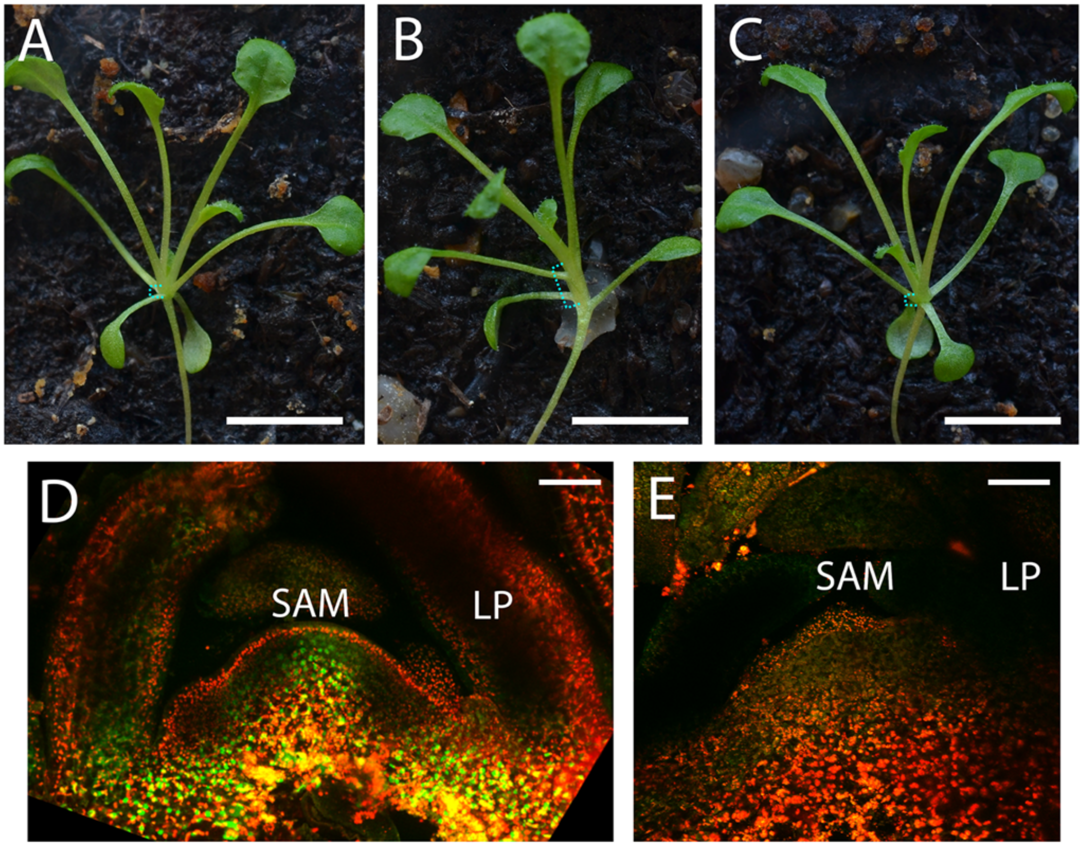

Fig. S1. Complementation of *ath1-3* with *pATH1::ATH1-GFP*

A-C: Wild-type Col (A), *ath1-3* (B) and *ath1-3 pATH1::ATH1-GFP* seedlings grown for 28 short days with EOD-FR treatment; compare the elongated internode in *ath1-3* with the region where internode growth is repressed in the wild type and in the complemented line (cyan brackets); bars: 5 mm. D,E: Maximum intensity projections of confocal image stacks through the apex of 21 day old *ath1-3 pATH1::ATH1-GFP* seedling (D) or the wild-type control (E); SAM and LP indicate the position of the shoot apical meristem and leaf primordia, respectively; note the expression of *pATH1::ATH1-GFP* (green signal in D) in the shoot meristem and near the base of developing leaves, against the autofluorescence background (red); scale bars: 50 μm..

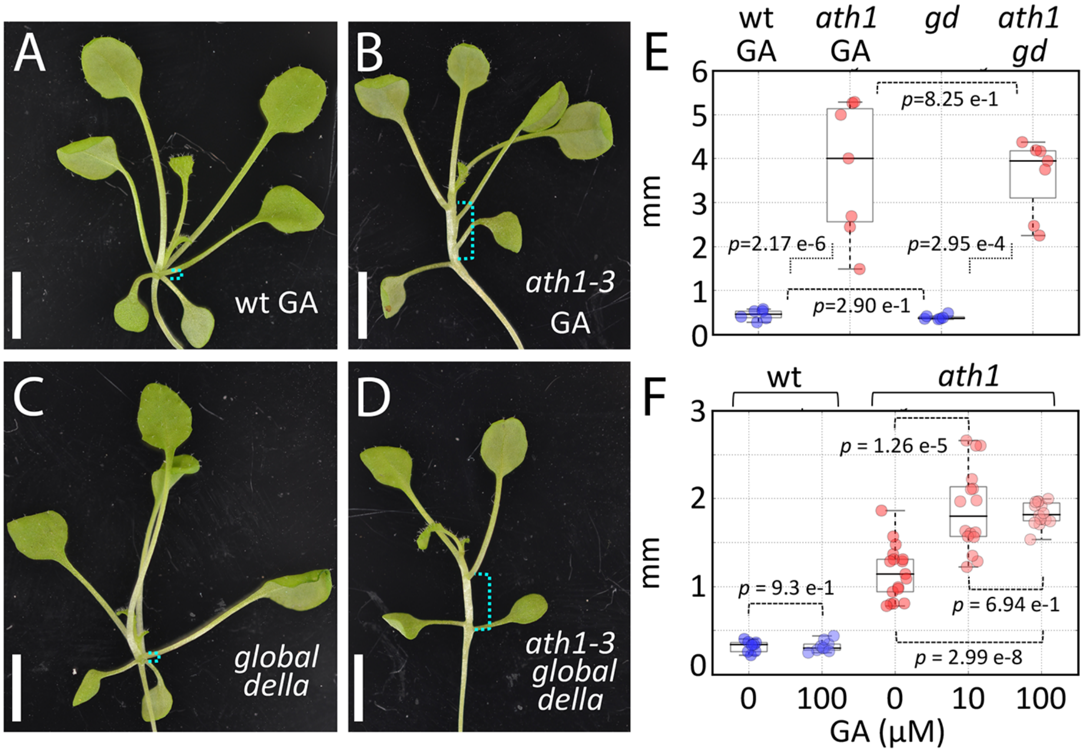

Fig. S2. GA treatment phenocopied the *ath1-3 global della* mutant

A-D: Representative seedlings grown for 21 days in short days supplemented with EOD-FR treatment; the wt (A) and single *ath1-3* mutant (B) were sprayed with 10 µM GA4 every three days (A,B); the *global della* mutant (C) and the *ath1-3 global della* (*gd*) mutant (D) were not sprayed (B); cyan brackets indicate the internode regions measured in E; bars: 5 mm. E : Length of the first two internodes internode of seedlings equivalent to those in A-D; *p*-values are for Welch's *t*-test; additional statistics are shown in Supplementary Table S4. F: Length of the first two internodes internode of wild-type and *ath1-3* seedlings grown in short days without EOD-FR treatment for 21 days and sprayed every third day with the indicated concentration of GA4; *p*-values are for Welch's *t*-test; see additional statistics in Supplementary Table S4.

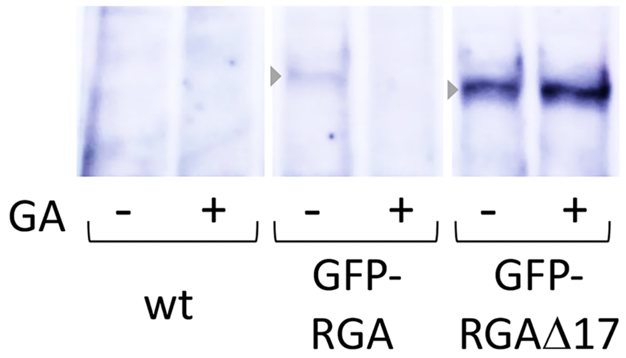

Fig. S3. Control experiment to confirm DELLA degradation in seedlings grown in medium with GA (as in Figure 5).

Western blot of whole protein from 21 days old seedlings grown on medium with GA4 10 μM or without GA, probed with anti-GFP antibody; wt was L-*er*; GFP-RGA and GFP-RGAΔ17, respectively, were transgenic lines with the *RGA1* promoter driving expression of a functional GFP-RGA fusion (*1*) or a GA-resistant version with the DELLA domain deleted (*2*); grey arrowheads indicate bands with the expected molecular size for GFP-RGA and GFP-RGAΔ17.

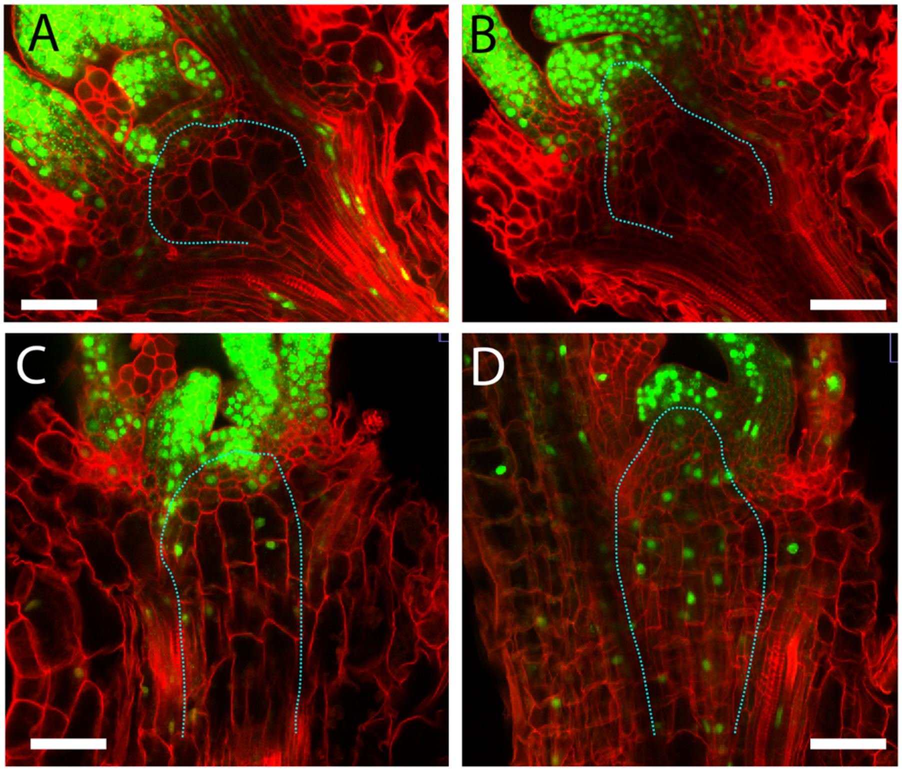

Fig. S4. Loss of ATH1 and DELLA function activates the vegetative rib meristem.

A-D: Optical sections through Arabidopsis seedlings grown for 10 short days, then labelled with EdU for 6 hours to mark cells that underwent DNA replication; cell outlines (labelled with propidium iodide) and EdU-labeled nuclei are shown in red and green, respectively; the dotted line in cyan indicates the rib meristem; A: wild-type; B: *global della* mutant; C: *ath1-3* mutant; D: *ath1-3 global della* sextuple mutant; images are representative of at least 4 biological replicates with comparable results for each genotype; bars: 50 µm.

Legends for Datasets S1 to S7:

Dataset S1 (separate file). List of ATH1 ChIP-seq targets.

Dataset S2 (separate file). GO analysis of ATH1 ChIP-seq targets (molecular function).

Dataset S3 (separate file). GO analysis of ATH1 ChIP-seq targets (biological process).

Dataset S4 (separate file). Raw data and statistical analysis for measurements of internode length.

Dataset S5 (separate file). Oligonucleotides used for qRT-PCR and genotyping.

Dataset S6 (separate file). Raw RT-qPCR data and statistical analysis.

Dataset S7 (separate file). ChIP-qPCR raw data and statistical analysis.

Supplementary references

1. A. L. Silverstone *et al.*, Repressing a repressor: Gibberellin-induced rapid reduction of the RGA protein in Arabidopsis. *Plant Cell* **13**, 1555-1565 (2001).
2. A. Dill, T. P. Sun, Synergistic derepression of gibberellin signaling by removing RGA and GAI function in Arabidopsis thaliana. *Genetics* **159**, 777-785 (2001).
